# Stellate ganglion blockade under ultrasound-guidance and the physiological responses in the rat

**DOI:** 10.3389/fphys.2024.1505038

**Published:** 2025-01-10

**Authors:** Robert M. N. Tran, Shaista Malik, Christopher Reist, Chad K. Oh, Najeebah Abdul-Musawir, Stephanie C. Tjen-A-Looi, Liang-Wu Fu, Theodore J. Baird, Anh T. Nguyen, Yiwei D. Gong, Zhi-Ling Guo

**Affiliations:** ^1^ Susan Samueli Integrative Health Institute and Department of Medicine, University of California, Irvine, Irvine, CA, United States; ^2^ AEON Biopharma, Inc., Irvine, CA, United States; ^3^ Department of Psychiatry, School of Medicine, University of California, Irvine, Irvine, CA, United States

**Keywords:** autonomic nervous system, heart, stellate ganglion, ultrasound, rat, lidocaine

## Abstract

Stellate ganglion blockade (SGB) is a practical approach to managing many clinical disorders. Ultrasound-guided SGB is currently adopted as a more effective and safer method in humans. Developing this technique in rats would facilitate further study of SGB application. The present study examined physiological responses following ultrasound-guided SGB in Sprague-Dawley rats. Under general anesthesia, lidocaine containing Chicago blue dye (1.0%–1.5% in 40–60 µL) was injected into the unilateral stellate ganglion (SG). Ptosis was observed on the ipsilateral right (n = 8) or left (n = 7) side of lidocaine administration. No ptosis was noted in any controls by 0.9% normal saline injection into the right (n = 6) or left (n = 6) SG. Heart rate (HR) was significantly decreased after administration of lidocaine (344 ± 32 to 289 ± 47 bpm; *p* = 0.015, pre-vs. after-injection), but not after normal saline, into the right SG. HR was unaltered after injecting lidocaine or normal saline into the left SG. Heart rate variability analysis showed that SGB with lidocaine on the right or left side caused a decrease in the ratio of the power of low-frequency over high-frequency. Respiratory rate, body temperature, and general conditions were unchanged in all rats, regardless of left or right SGB. Chicago blue dye was confirmed to be distributed in the SG region. No bleeding or tissue damage was evident in the injected SG area. Our findings suggest that ultrasound-guided unilateral SGB effectively inhibits cervicothoracic sympathetic nerves in rats and enhances heart rate variability, and sympathetic nerves controlling HR are likely predominantly associated with the right SG in the rat.

## Introduction

The stellate ganglion (SG) in humans is a series of sympathetic nerves formed by the fusion of the inferior cervical ganglion and the first thoracic sympathetic ganglion. The SG is an essential component of the sympathetic nervous system and the primary source of sympathetic nerves distributed to the head and upper body (Aleanakian et al., 2020; Li J. et al., 2022). Conducting SG blockade (SGB) via local anesthetics is a practical approach used to manage many clinical conditions, including regional pain syndrome associated with the head, neck, and upper extremities, craniofacial hyperhidrosis, refractory angina, vascular headaches, hot flashes, post-traumatic stress disorder, and depression (Aleanakian et al., 2020; Othman and Zaky, 2014; Rae Olmsted et al., 2020). Local anesthetics commonly used for SGB at present include lidocaine, bupivacaine ropivacaine, and levobupivacaine. These anesthetics diffuse and bind to sodium ion channels to prevent depolarization and thus inhibit nerve electrical conduction. The rat’s SG is composed of the inferior cervical ganglion fused with the first three thoracic ganglia (Abdi and Yang, 2005; Baron et al., 1995). While smaller, it is anatomically similar to the human SG (Abdi and Yang, 2005; Baron et al., 1995). Therefore, the rat is considered a useful model for investigating SGB (Abdi and Yang, 2005; Dai et al., 2021; Li T. T. et al., 2022; Lin et al., 2022).

SGB performed in humans typically relies solely on body landmarks (e.g., C7 transverse process) identified manually or by X-rays and lacks visualization of the interior structures or needle path. Not doing so can lead to complications caused by injuries to various vital structures around the SG, including pneumothorax, puncturing of neighboring vasculature or nerves such as the carotid artery, internal jugular vein, vagal nerve, and brachial plexus roots. Fluoroscopic guided-SGB relies on X-rays to visualize the interior anatomy, inferring from visible bones, but does not allow easy visualization of soft tissues and blood vessels (Aleanakian et al., 2020; Li J. et al., 2022). Ultrasound-guided SGB is currently being adopted as a more effective method to reduce adverse reactions and complications in clinical applications. It allows for visualization of tissue and blood vessels and identification of SG by its surrounding structures. In addition, ultrasound has a better safety profile since sound waves are non-ionizing. Because the subjects suffer almost no adverse effects from the ultrasound waves, they recover quickly after the procedure (Aleanakian et al., 2020; Li J. et al., 2022). More importantly, it lets the operator clearly see the structures around SG and the injecting needle. This clinical applicability is why ultrasound is becoming the preferred imaging modality for SGB.

Radiation raises concerns in developing SGB techniques for rodents, as several studies have shown that rats can perceive radiation and that continuous exposure can cause behavioral anomalies (Mantraratnam et al., 2022). Alternative approaches for SGB in the rat are to use a thoracic surgical procedure to gain direct access and visualize the SG or a blind injection approach (Abdi and Yang, 2005; Gulcu et al., 2009; Kreusser et al., 2006). However, the efficacy of SGB with a blind injection approach remains unclear, while the adverse effects of these methods pose significant concerns (Gulcu et al., 2009; Lin et al., 2022). In this regard, one prior study reported that decreased heart rate (HR) was observed with bilateral SGB but not unilateral SGB using a blind injection of 0.25% bupivacaine with a large volume (0.2 mL) (Abdi and Yang, 2005). This observation is inconsistent with clinical application as bilateral SGB is rarely used. Also, concern was raised about whether unilateral SGB could effectively reduce cardiac sympathetic nerve activity using a more appropriate approach. Recently, Lin et al. were the first to test left SGB under ultrasound guidance in rats and evaluated the efficacy only by identifying ptosis resulting from blocking the oculosympathetic pathway. They did not examine HR, other physiological responses, and the rat’s activity in conscious condition following SGB (Lin et al., 2022).

From a clinical standpoint, the right-sided SGB is the standard practice for managing some clinical disorders, such as post-traumatic stress disorder, depression, and chronic stress (Hanling et al., 2016; Rae Olmsted et al., 2020; Wang et al., 2017). For the reasons mentioned above, in the present study, we endeavored to evaluate further the feasibility, physiological effects, and safety of using ultrasound to guide SGB on each side (right or left) in the rat based on Lin’s report on left SGB. The procedure’s efficacy and safety profiles were assessed by analysis of potential changes in HR, HR variability (HRV), respiration, temperature, clinical signs of blockade of sympathetic pathways (e.g., ptosis), general status, and tissue damage relevant to the procedures’ target (SGB) and route to that target. HRV is used to indicate indirectly the status of autonomic nervous activity, particularly relevant to the heart. The inhibition of cardiac sympathetic nerve activity is associated with decreased HR and increased HRV (Li and Zheng, 2022; Matouk et al., 2018; Švorc et al., 2023). The inhibition of sympathetic nerve activity slows the respiration (Servant et al., 2009). There is no information available on the influence of SGB on HRV and respiratory rate in rats. Thus, we hypothesized that ultrasound-guided unilateral SGB effectively inhibits cervicothoracic sympathetic nerves in the rat, leading to reductions in HR and respiratory rate with an increase in HRV.

## Materials and methods

Male Sprague Dawley rats (310–350 grams) were obtained from Charles River Laboratories. The rats were housed in two per cage and received a standard food and water diet *ad libitum*. Conditions were controlled to keep the room on a standard 12-h light/dark cycle and the temperature at 22°C ± 2°C. The study conformed to the American Physiological Society’s Guiding Principles for Research Involving Animals. All the procedures in this experiment were approved by the Institutional Animal Care and Use Committee of the University of California Irvine.

### Experimental protocols

Rats were anesthetized initially using 4% isoflurane mixed with oxygen in an induction chamber (Visualsonics Vevo Compact Anesthesia) and then transferred to the animal monitoring station (Vevo SR200). Anesthesia was maintained with 2%–3% isoflurane through a nosecone attached to the monitoring station. The rats were then secured supine to the station’s electrodes with electrode gel (Parker Laboratories) applied beneath the paws, which allowed electrocardiogram (ECG) at leads I, II, and III to be monitored and recorded. Core temperature was measured using an integrated rectal probe while ECG at lead II, HR, and respiratory rate were monitored in real-time using built-in electrodes on an animal monitoring platform (Vevo SR200). This platform allowed us to adjust the heating to preserve the body temperature within the normal physiological ranges during the SGB procedures. Constant anesthesia was kept and judged by normal and stable HR, respiration, body temperature, and the lack of responses to corneal probing and paw pinch.

**FIGURE 1:**
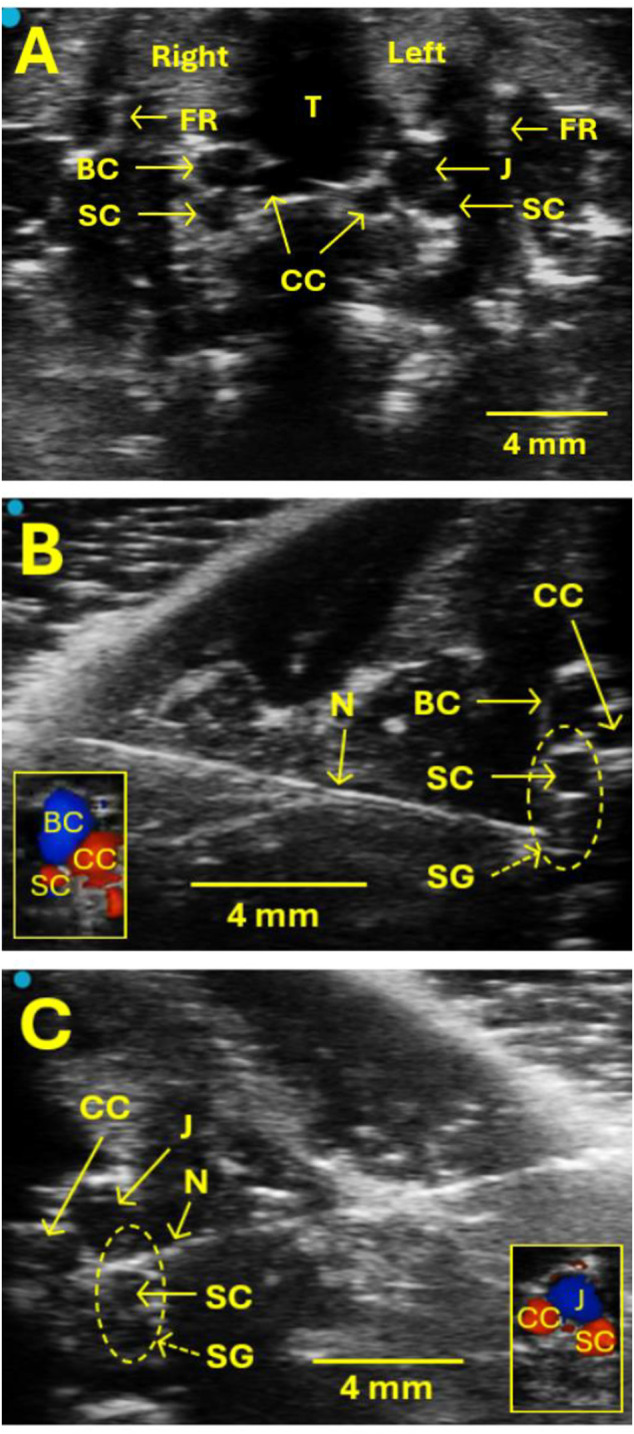
Ultrasound images show the location of the SG for the injection. **(A)** The SG itself is not visible on ultrasound, but some valuable landmarks help locate SG and guide the SG injection safely without puncturing blood vessels. These landmarks include the trachea (T), the first rib (FR), the common carotid artery (CC), the brachiocephalic vein (BC), the jugular vein (J), and the subclavian artery (SC). The SG is located near SC on both the right and left sides. **(B, C)** Examples of images showing the SG injection on the right **(B)** and left **(C)** sides, respectively. Vertical oval areas formed by dot lines in B and C indicate SG locations. N indicates a needle. CC, BC, J, and SC were verified by Doppler examination, as illustrated in the small box in the lower left corner **(B)** and right corner **(C)**.

The regions around the clavicle and chest were shaved and cleaned. The skin was disinfected with a chlorhexidine disinfectant. The linear ultrasound transducer (MX250, FUJIFILM VisualSonics, Vevo 3100 LT imaging system) was placed at the heart’s position in the short axis view and moved anteriorly until the trachea, first rib, common carotid artery, the brachiocephalic or jugular vein, and subclavian artery could be seen (Figure 1A). These structures served as landmarks to help locate SG. SG was not visible but was located based on its surrounding structures mentioned above using ultrasound. In this respect, the SG is near the subclavian artery on both the right and left sides. The animal monitoring platform was integral in maintaining proper positioning for the ultrasound transducer and allowed for image fine-tuning (Vevo SR200).

After establishing a stable baseline of ultrasound monitoring for a 10-min interval, a 26-gauge hypodermic needle with a 1 mL syringe (marked every 10 µL) was advanced in-plane with the transducer from lateral to medial to the SG region (Figures 1B, C). At the proper injection site of the SG, the transducer should be in close proximity to the collarbone since SG is near the first rib. This served as an additional method to confirm the SG’s correct injection site. When the needle tip reached the target SG region, the syringe cylinder was withdrawn slightly to verify that no blood was present due to the procedure as executed to that point. A single injection of 1.0%–1.5% lidocaine or 0.9% normal saline mixed with Chicago blue dye at 40–60 µL was administered to the right or left SG. The administration of injected liquid was observed, and the injection process was monitored directly with ultrasound. After the administration, the needle was removed.

The ECG (lead II), HR, respiration, and temperature were monitored in real-time and recorded 10 min before and after the SG injection for retrograde analysis. Afterward, rats were put back in the cage and allowed to recover from the anesthesia. Recovery was commonly observed 2–5 min after terminating the anesthesia. Once the rat recovered, as justified by the free movement in the cage, the eyelid status (presence and degree or absence of ptosis) and general conditions were closely monitored for 60 min, then hourly for 6 hours following the SG injection. The rat activity was evaluated by observing the magnitude of overall movement. Ptosis was assessed by comparing the degree of dropping of the eyelid after the SG injection to pre-injection on the ipsilateral eye as well as on the contralateral eye following the SG procedure. The degree of ptosis was identified using a scale of 1-4 in increments of 25% eyelid closure, representing 25%, 50%, 75%, and 100% eyelid drooping, respectively. An observation of 100% eyelid drooping was the same as the wholly closed eyelid.

At the end of the experiment, rats were cannulated with a catheter through a femoral vein under anesthesia with 4% isoflurane. 1M potassium chloride (0.5 mL) was administered intravenously to euthanize rats humanely. Afterward, the rat’s chest was opened. The SG region was grossly examined at the base of the first rib beneath the prevertebral fascia between the first and second ribs (Kreusser et al., 2006). Proper labeling of the SG with blue dye was confirmed.

### Experimental groups

Rats were randomly divided into four groups, including right SG lidocaine injection (n = 8), right SG 0.9% normal saline injection (n = 6), left SG lidocaine injection (n = 7), and left SG 0.9% normal saline injection (n = 6).

### Data analysis

The ECG, HR, respiratory rate, and temperature were recorded by the monitoring station (Vevo SR200), and the tablet interface processed the recording data (Vevo Monitor app). Ultrasound images were analyzed in a separate specialized program (Vevo Lab) that allowed for more detailed analysis of the images. Data of ECG at lead II from the monitoring station were uploaded into HRV software (Kubios, version 3.5.0; Kubios Ltd.) to generate a graphical representation of HR tracings and to analyze HRV.

Frequency domain analysis was performed to examine the cardiac sympathetic and parasympathetic balance using Kubios HRV software. A power spectrum density estimate was calculated for the RR interval series. The spectrum was estimated using a Fast Fourier transformation (FFT) method with Welch’s periodogram. The cut-off frequencies for the power spectral analysis included total power frequency (0.01–3.00 Hz), very-low frequency (VLF, 0.01–0.20 Hz), low frequency (LF, 0.20–0.80 Hz), and high frequency (HF, 0.80–3.00 Hz). LF and HF values in normalized units (nu) indicate the relative power of the low-frequency band and high-frequency band, respectively, to the power value derived from total power minus the power of the very low-frequency band [i.e., LF or HF/(Total power–VLF) × 100] (Li and Zheng, 2022). LF and HF values (nu) were commonly used to reflect main sympathetic and parasympathetic-related activities, respectively, although some studies suggest that LF may represent both sympathetic and parasympathetic activities. The LF/HF ratio was calculated to indicate sympathovagal balance, which is widely used and accepted (Pichot et al., 2016; Švorc et al., 2023).

Data are expressed as means ± SD. The Shapiro-Wilk test was used to determine if the data were normally distributed. Comparisons between the two groups were statistically analyzed using the Student's t-test and Mann–Whitney rank sum test. A P-value of less than 0.05 was considered significant.

## Results

### Changes in HR in response to SGB

**FIGURE 2:**
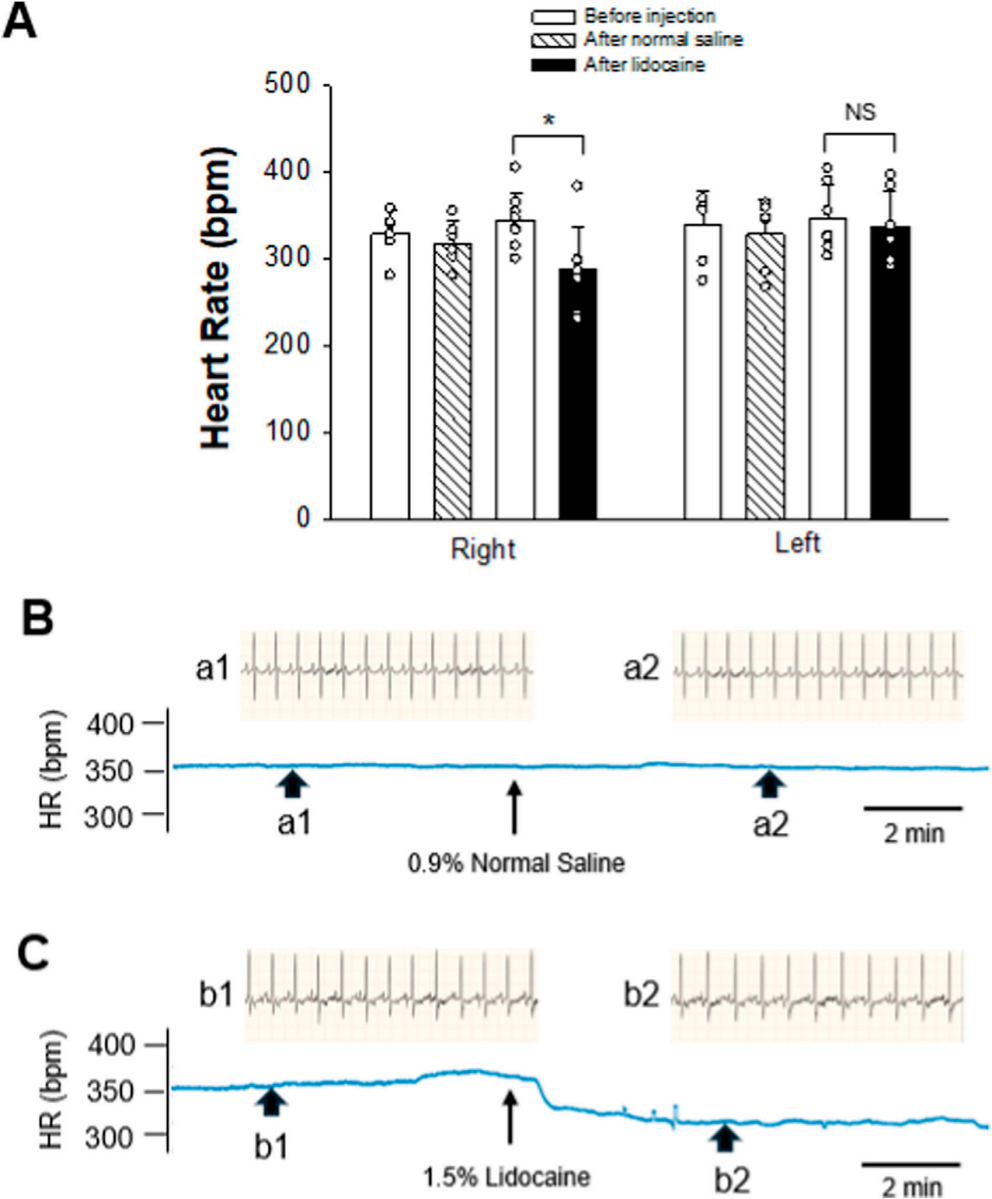
Changes in HR after intervening SG. **(A)** graphs show group data of SG blockade by 1.0%–1.5% lidocaine on either the right (n = 8) or left (n = 7) side. In controls, 0.9% normal saline was administered into the SG on either the right or left side (n = 6 on each side). Data are expressed as means ± SD. Comparisons between the two groups were statistically analyzed using the Student’s t-test and Mann–Whitney rank sum test. * indicates P < 0.05 for after vs. before the SG injection of either lidocaine or normal saline. NS indicates no significant difference. There was no significant difference in HR baselines prior to the application of lidocaine or normal saline into SG on the same right or left side. Also, the HR baselines before administration of lidocaine or normal saline into SG were not different between the right and left sides. **(B, C)** Diagrams displaying examples of the change in HR after intervening SG. HR tracings derived from the recording of ECG before and after administering either 0.9% normal saline **(B)** or 1.5% lidocaine **(C)** in volumes of 40 µL on the right SG. Arrowheads below the HR tracing indicate the site where examples of original ECG were taken. Labels of the arrowheads, a1, a2, b1, and b2, correspond to those indicating original ECG recordings over 2.2 s before (a1 and b1) and after (a2 and b2) the SG intervention, displayed above the HR tracing. Arrows below HR tracings in **(B, C)** indicate the time of the SG injection.

HR baseline (10 min) was stable and similar across all groups, as shown in Figure 2. There were no significant differences in HR baseline between experimental groups. The injection of 40–60 µL of 0.9% normal saline into the right SG did not alter HR. However, applying 1.0%–1.5% of lidocaine at 40–60 µL into the right SG caused a significant decrease in HR from 344 ± 32 to 289 ± 47 bpm (*p* = 0.015, n = 8; Figure 2A), lasting for at least 10 min. Figures 2B, C show examples of changes in HR following the administration of normal saline and lidocaine into the right SG. HR was not changed significantly after injecting either 0.9% normal saline or 1.0%–1.5% lidocaine at 40–60 µL into the left side SG.

### Alterations in HRV induced by SGB

**FIGURE 3:**
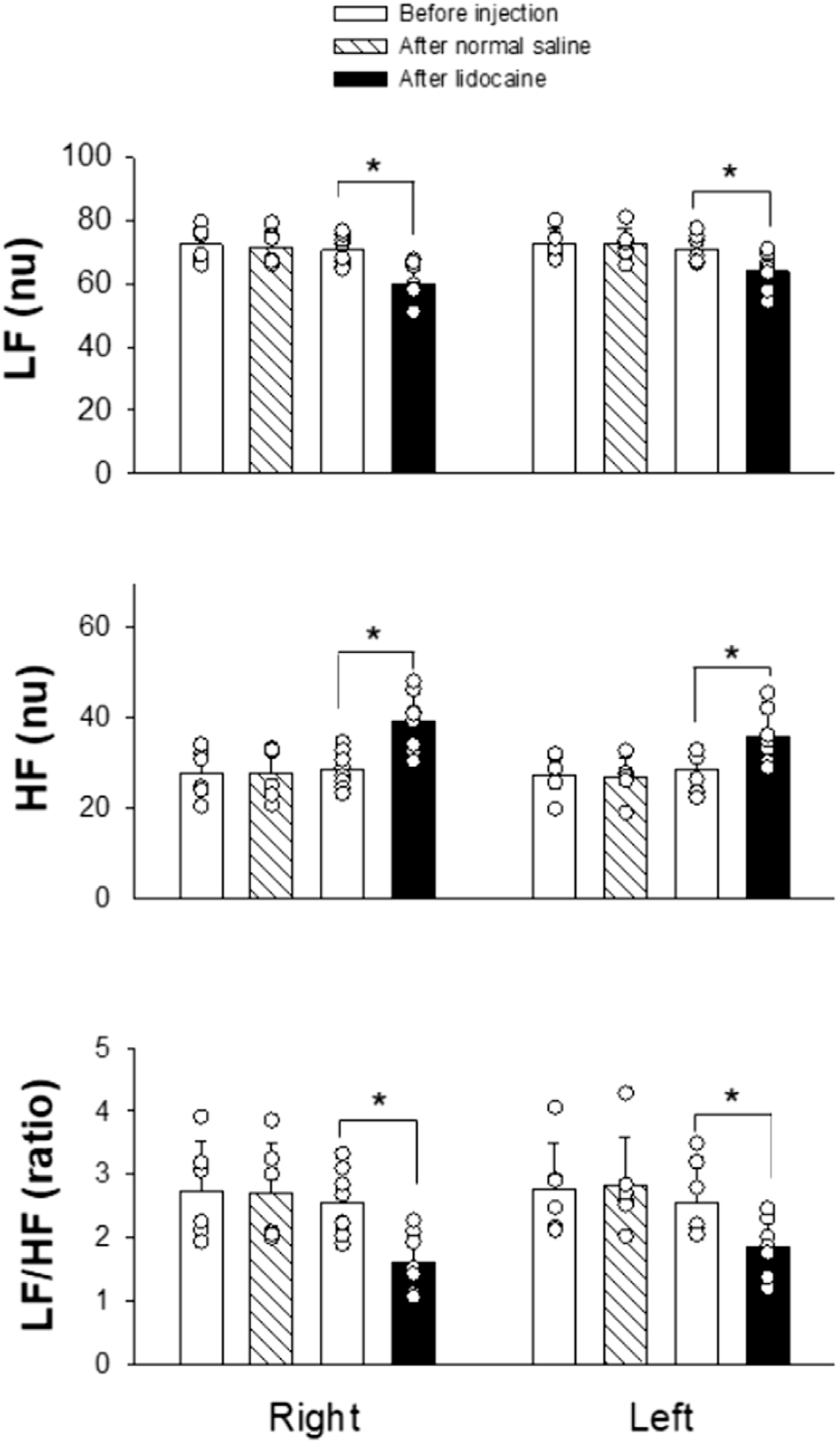
Changes in HRV after intervening SG. The SG was blocked by 1.0%–1.5% lidocaine on either the right (n = 8) or left (n = 7) side. In controls, 0.9% normal saline was administered into the SG on either the right or left side (n = 6 on each side). Data are expressed as means ± SD. Comparisons between the two groups were statistically analyzed using the Student's t-test and Mann–Whitney rank sum test. * indicates P < 0.05 for after vs. before the SG injection of either lidocaine or normal saline. There was no significant difference in the measurement before applying lidocaine or normal saline into SG on the same right or left side. Also, the values before administration of lidocaine or normal saline into SG were not different between the right and left sides. HF, high frequency; LF, low frequency; nu, normalized units.

We measured HRV using frequency domain analysis before and after SGB. Power spectral analysis of the HRV components allows separate estimation of sympathetic and parasympathetic effects of SG treatment on the heart. As demonstrated in Figure 3, injecting normal saline into either the right or left SG did not alter the power values of LF (nu), HF (nu), and the ratio of LF/HF. In contrast, administering 1.0%–1.5% lidocaine into SG on the right side (n = 8) significantly caused a decrease in LF (from 70.52 ± 4.80 to 59.84 ± 6.47 nu; *p* = 0.002), an increase in HF (from 28.45 ± 4.04 to 39.05 ± 6.40 nu; *p* = 0.001), and a decrease in the LF/HF ratio (from 2.54 ± 0.52 to 1.60 ± 0.45; *p* = 0.002). Similarly, 1.0%–1.5% lidocaine injected into the left SG (n = 7) significantly decreased LF (from 70.86 ± 4.50 to 63.98 ± 6.09 nu; *p* = 0.033), increased HF (from 28.56 ± 4.45 to 35.81 ± 6.06 nu; *p* = 0.025), and decreased the LF/HF ratio (from 2.56 ± 0.59 to 1.85 ± 0.46; *p* = 0.029). A marked decrease in the LF/HF ratio was attributed to a reduced LF component and enhanced HF component. Generally, HF reflects cardiac parasympathetic activity, while LF and LF/HF ratio reflect cardiac sympathetic modulation. These data indicate an increase in HRV induced by right or left SGB, which was likely associated with suppressed sympathetic activity and augmented parasympathetic tone. As such, at least partially, the findings reflect a reduction in the sympathetic prevalence in rats treated with SGB.

### Responses of respiratory rate and temperature following SGB

We observed no abnormal respiration patterns and rhythms qualitatively in any rat. The respiratory rate underwent minimal changes as either lidocaine or normal saline was administered into either the right or left SG. There were no significant differences in respiratory rate between pre- and post-injections of any testing agent in any experimental groups (Supplementary Table S1). In this regard, especially, we noted that the respiratory rate was from 54 ± 19 to 56 ± 13 breaths per minute (n = 8; *p* = 0.868) in rats subjected to lidocaine application into SG on the right side. Similarly, the respiratory rate was from 53 ± 15 to 58 ± 13 breaths per minute (n = 7; *p* = 0.469) in rats that underwent lidocaine administration into the left SG.

The core body temperature of the rats did not change significantly for any experimental group (Supplementary Table S1). Administration of lidocaine or normal saline into either the right or left SG did not significantly alter the core temperature. In particular, we noticed that the body temperature at pre- and post-injection of lidocaine into SG on the right or left side was 36.21 ± 0.34 vs. 36.19°C ± 0.37°C or 36.44 ± 0.39 vs. 36.49°C ± 0.38°C (before vs. after injection; all *p* > 0.05), respectively.

### Appearance of ptosis after SGB

**FIGURE 4:**
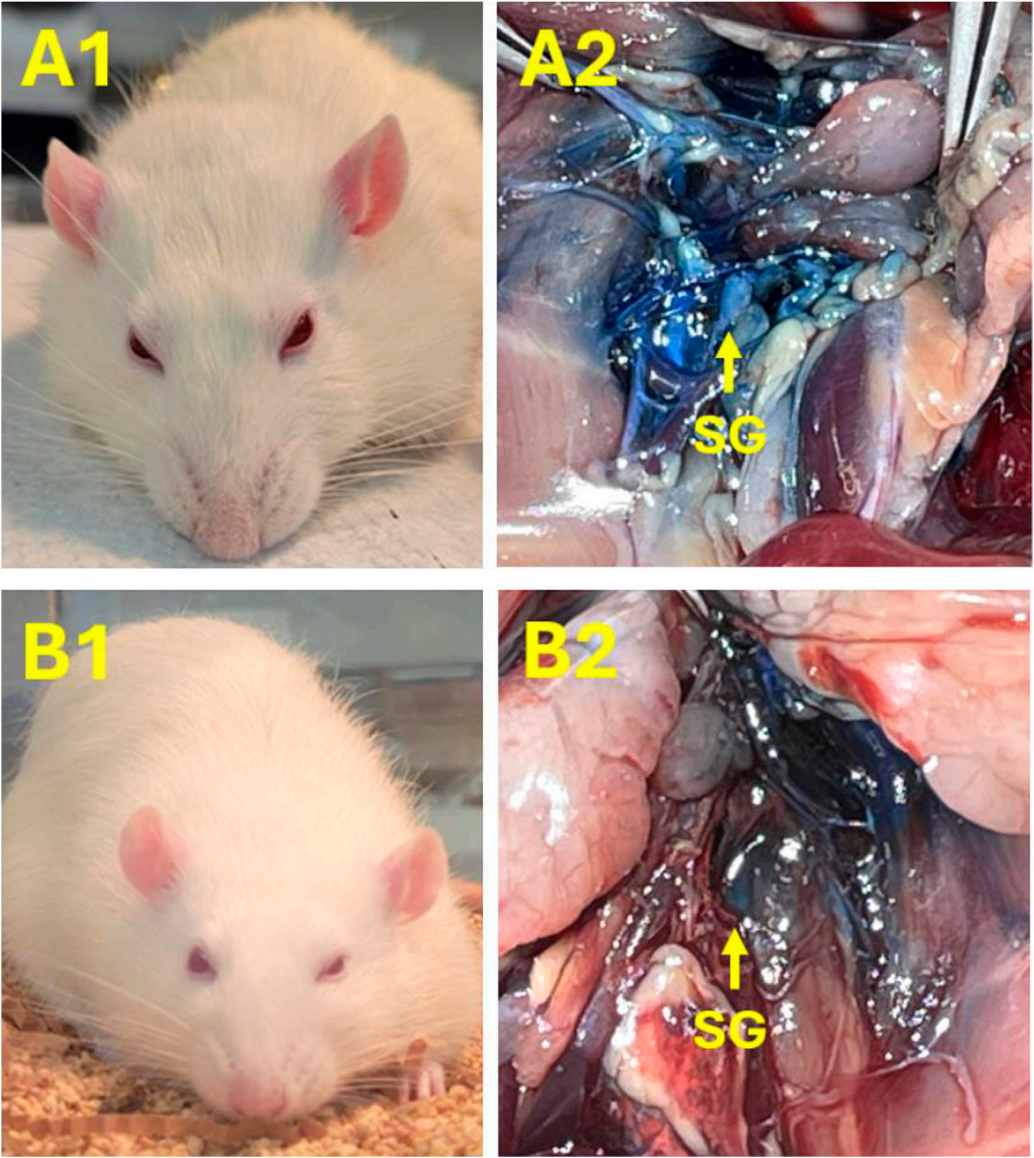
Images demonstrating examples to verify successful SGB with lidocaine on the right or left side. **(A1, B1)** Ptosis occurred in rats following SGB with 1% lidocaine on the right [60 μL; **(A1)**] or left [40 μL; **(B1)**] sides, respectively. Ptosis did not appear in any rat after injecting 0.9% normal saline in the SG. Panel A2 and B2: SG labeled with the blue dye further verified the correct SG injection site on the right **(A2)** and left **(B2)** sides, accordingly.

All of the rats recovered from the general anesthesia within 2–5 min and moved in the cage freely. We noted that ptosis appeared on the ipsilateral, but not the contralateral, side of lidocaine injection, whether directed at the right or left SG (Figure 4). In addition, it was observed that ptosis ranged from Grade 2 to 4 (e.g., 25%–100% of eyelid drooping; Supplementary Table S2). The timeframes for ptosis duration ranged from 15 to 40 min. In contrast to those animals treated with lidocaine, ptosis was not observed in any rats subjected to the injection of normal saline into the right or left SG (Supplementary Table S2).

### General conditions following SGB

After recovering from applying lidocaine or normal saline into the right or left SG under general anesthesia, rats’ conditions were observed closely for 6 hours. A slight decrease in activity was noted immediately following recovery from anesthesia compared to before the SG injection. Those rats who underwent lidocaine administration appeared to have less movement than those who received normal saline within about 1 hour after the SG injection. Afterward, all rats reverted to normal levels of activity. In this regard, rats moved normally with normal levels of eating and drinking. All animals also appeared normal in terms of urinary and excretory function. No rats were observed to be moribund or died following the SG injection procedure.

### Gross macroscopic findings at the site of SGB

After opening the chest, SG, a star-shaped structure, was identified between the first and second ribs beneath the prevertebral fascia in the area near the lower edge of the first rib. The SG was located near the subclavian artery and anterior or immediately lateral to the longus colli muscle. The SG lay just anterolateral to the seventh cervical vertebral body at the base of the C7 transverse process, lateral to the first thoracic vertebral body, and in the groove between the vertebral body and the transverse process. The SG region was highlighted as a blue-white hue from the Chicago Blue dye mixed with the injected agent (Figure 4). This was observed in all rats and, as such, represented a definitive visual confirmation that the SG injection occurred at the correct site. There were no signs of bleeding or significant tissue damage in the SG region and its surroundings.

## Discussion

The present study aimed to examine potential physiological changes in response to unilateral SGB under ultrasound guidance in rats. Changes in HR induced by bilateral SGB were previously reported using a blind injection approach in rats, which is not a practical protocol with frequent occurrences of complications (Abdi and Yang, 2005). One recent study introduced SGB under ultrasound guidance in rats but mainly focused on the left side and did not make HR and other physiological observations (Lin et al., 2022). Using the ultrasound technique and an advanced monitoring station, the present study showed that ultrasound-guided SGB with lidocaine on the right side, but not the left side, significantly decreased HR in the rat. HRV analysis showed that SGB on the right or left side caused a decrease in LF, an increase in HF, and a decrease in the LF/HF ratio, suggesting that unilateral SGB may enhance HRV and modulate cardiac sympathetic activity and sympathovagal balance. Unilateral SGB on either side did not alter the respiratory rate, core body temperature, and general status. Ptosis consistently appeared on the ipsilateral side following unilateral SGB, indicating the successful execution of SGB. A gross examination showed the presence of dye but the absence of bleeding or tissue damage in the injected SG area. Although the results did not support our working hypothesis fully, our findings suggest that ultrasound-guided unilateral SGB can effectively block cervicothoracic sympathetic nerves in rats, particularly influencing cardiac sympathetic activity.

Building on one previous study using left SGB under ultrasound guidance (Lin et al., 2022), the present study confirms that the ultrasound-guided SGB method allows for a precise approach and targeting of the SG location on either right or left SGB while avoiding damage to adjacent vital blood vessels and soft tissues. Tissue landmarks, including the subclavian artery and the brachiocephalic and jugular vein, could be visualized, allowing the correct SG area to be located and the needle to be properly positioned. In addition, this precise targeting allows for smaller doses (40–60 µL) of nerve block agent to be used. It is an advantage over blind injection procedures where large injection volumes (200 µL) are needed to achieve satisfactory effects in rats (Abdi and Yang, 2005; Dai et al., 2021; Gulcu et al., 2009; Wang et al., 2017). Having demonstrated the feasibility and physiological effects, future investigators wanting to study the role of SG in the sympathetic nervous system can be confident in this method.

SGB is known to result in Horner’s Syndrome, including ptosis, miosis, enophthalmos, and anhidrosis. This is partly mediated by inhibiting sympathetic input to the oculosympathetic pathway. These signs are commonly used in the clinic to ascertain the successful execution of SGB in humans (Aleanakian et al., 2020; Li J. et al., 2022). Consistent with previous studies (Abdi and Yang, 2005; Gulcu et al., 2009), we noted that it was difficult to measure anhidrosis, miosis, and enophthalmos in rats reliably. As such, ptosis was used as the primary marker to assess the success of SGB. We observed ipsilateral ptosis consistently after unilateral application of lidocaine but not normal saline. The consistency of these observations strongly indicated that SGB was successful and, accordingly, that the technique of ultrasound-guided SG injection was reliable.

The heart is directly regulated by the sympathetic nervous system and would be expected to be affected by SGB. However, the effect of unilateral SGB on HR has been inconsistent. In this respect, some human studies using a blind method of SGB showed that unilateral SGB induces a decrease (Tarazi et al., 1978), an increase (Ikeda et al., 1996), or no apparent changes in HR (Fujiki et al., 1999; Gardner et al., 1993; Puente de la Vega Costa et al., 2016). One possible explanation was that the local anesthetic solution injected into the SG might spread to surrounding areas and block vagal nerves adjacent to the SG (Ikeda et al., 1996). Other clinical studies demonstrated that a decrease in HR resulted only from the right but not the left SGB (Kashima et al., 1981; Rogers et al., 1978), suggesting that right and left SGB affect HR differently. The inconsistency of these previous observations might be due to the inherent inaccuracy in the blind technique. The effect of SGB on HR in animals is also unclear. Blind SG unilateral injection (both left and right) with 0.25% bupivacaine did not lower HR, but bilateral SGB did (Abdi and Yang, 2005). It is not easy to reconcile these findings, but the possibility exists that SGB was not consistently achieved. In our study, where SGB was achieved with high certainty, we found that SGB with lidocaine on the right side decreased HR. The reduced HR was still in the normal physiological range for the rat (250–493 bpm) (Milani-Nejad and Janssen, 2014). In contrast, there was a lack of any meaningful changes in HR in response to the sham injection of 0.9% normal saline, the vehicle for lidocaine. In addition, we noted that left SGB yielded no significant reduction in HR, although the successful execution of SGB was confirmed by ipsilateral ptosis and histological verification. These results imply that right and left SGB have different impacts on HR in the rat, which is likely caused by unequal innervation of the sympathetic nerves on the heart from each side of SG (Rogers et al., 1978; Yanowitz et al., 1966). Efferent sympathetic nerves from the left SG are distributed to the atrioventricular node and the posterior myocardium of the left ventricle. In contrast, those from the right SG are distributed in the sinus node and the anterior myocardium of the left ventricle (Rogers et al., 1978; Yanowitz et al., 1966). As such, the HR is likely more influenced by efferent sympathetic innervation from the right SG. The decrease in HR induced by the right SGB may serve as a sign of a successful right SGB, particularly in rats under anesthesia.

To elucidate further SGB’s effect on cardiac autonomic function, we measured HRV before and after SGB. To our knowledge, this is the first study to examine changes in HRV by SGB in rats. We observed that unilateral right or left SGB induced a decrease in LF, an increase in HF, and a decrease in the LF/HF ratio. While HF is agreed to be mainly related to vagal activity, disagreement exists regarding the LF components. Some studies consider LF to be relevant to both sympathetic and parasympathetic activities. Other studies suggest that the LF mainly reflects the status of the sympathetic nerve when it is expressed in nu. Many investigators commonly combined the LF with the LF/HF ratio to highlight sympathetic modulations or alterations of sympathovagal balance (Li and Zheng, 2022; Matouk et al., 2018; Švorc et al., 2023). Parallel decreases in LF and the LF/HF ratio following SGB likely indicate a reduction in cardiac sympathetic nerve activity or the ratio between sympathetic and parasympathetic nerve activities. Reduced sympathetic tone reflected by HRV analysis would be consistent with the effects of SGB on sympathetic pathways that mediate ptosis. We noted no marked alteration in HR, but HRV following the left SGB, compared to significant changes in both HR and HRV after the right SGB. As previously discussed, it is likely that HR is less influenced by efferent sympathetic innervation from the left SG, which is not primarily distributed to the heart’s sinus node. The increased HF following either right or left-sided SGB may be a consequence of unopposed parasympathetic nerve activity due to decreased sympathetic nerve activity. In support of our explanation, Saxena et al. reported a case of sinus arrest after SGB. They suggested that sympathetic nerve block after SGB led to vagal accentuation to trigger asystole (Saxena et al., 2004). The exact mechanisms underlying these observations need to be further investigated. Our HRV data support the conclusion that the ultrasound-guided SGB can effectively block cardiac sympathetic nerves while likely avoiding inhibition of vagal nerves, which can happen using the blind method of SGB (Fujiki et al., 1999; Ikeda et al., 1996).

The SG is believed to regulate the systemic autonomic nervous system by influencing central brain regions, mainly the hypothalamus, to maintain homeostasis (Rae Olmsted et al., 2020; Wang et al., 2017). Peripherally, SG participates in regulating the relaxation and contraction of bronchial smooth muscle (Brookhart et al., 1936; Chen et al., 2016; Othman and Zaky, 2014). We noted, however, that unilateral SGB did not impact respiration and core temperature. In support of our observations, one previous study in dogs showed that the SGB did not influence the respiratory system under normal conditions (Okuda et al., 1995). No information is available on the changes in body temperature caused by SGB in normal status. As such, our results suggest that unilateral SGB, as executed in this study, does not represent a strong enough manipulation to inhibit the sympathetic activity associated with respiration and core temperature in normal conditions.

The present study showed that rats appeared to move less within the first hour of recovery post-injection of lidocaine, thereafter returning to normal activity levels. SGB is known to reduce sympathetic inputs to the brain, and such sympathetic inhibition induced by SGB may mitigate activity associated with stressful states in rats similar to that known to occur in humans (Lynch et al., 2023; Rae Olmsted et al., 2020). Overall, all rats showed no significant changes in activity and no complications after the injection procedure. Those negative changes support the safety of ultrasound-guided SGB.

### Limitations of the study

The potential limitation of this study was that the SG injection was conducted under anesthesia since significant levels of rat movement did not allow for the SG injection to be performed safely and effectively in a conscious condition, even if the rat was restrained. Moreover, restraint is well-known to induce significantly greater stress. However, the ultrasound-guided SG injection procedure conducted under anesthesia allowed the evaluation of this method effectively in rats, and it better characterized the immediate responses to SG injection compared to sham-operated control conditions. These findings help us understand ultrasound-guided SGB and relevant acute physiological responses. The later observations in conscious status after the SG injection allowed us to assess the adverse effects and safety of SGB as it happens in humans.

## Conclusion

Noninvasive ultrasound-guided unilateral SGB can effectively block cervicothoracic sympathetic nerves, especially leading to a decrease in HR and a reduction in LF/HF ratio in HRV. These results support the utilization of this approach to further understand mechanisms underlying the use of SGB in rat disease models.

## Data Availability

The original contributions presented in the study are included in the article/Supplementary Material, further inquiries can be directed to the corresponding author.

## Abbreviations

ECG, electrocardiogram; HR, heart rate; HRV, heart rate variability; HF, high frequency; LF, low frequency; nu, normalized units; SG, stellate ganglion; SGB, stellate ganglion blockade.
