# Supplementary material for: Stellate ganglion blockade under ultrasound-guidance and the physiological responses in the rat
Source: Front Physiol. 2025 Jan 10;15:1505038. doi: 10.3389/fphys.2024.1505038 (PMC11757301; doi:10.3389/fphys.2024.1505038)
Supplement: Supplementary file 1 [file Table1.docx]

***Supplementary Materials***

**Stellate Ganglion Blockade under Ultrasound-Guidance and the Physiological Responses in the Rat**

Robert M. N. Tran^1^, Shaista Malik^1^, Christopher Reist^2, 3^, Chad K. Oh^2^,

Najeebah Abdul-Musawir^2^, Stephanie C. Tjen-A-Looi^1^, Liang Wu Fu^1^, Theodore J. Baird^2^, Anh T. Nguyen^1^, Yiwei D. Gong^1^, Zhi-Ling Guo^1^*

^1^Susan Samueli Integrative Health Institute and Department of Medicine, University of California, Irvine at Irvine, CA, USA

^2^AEON Biopharma, Inc., Irvine, CA, USA

^3^Department of Psychiatry, School of Medicine, University of California, Irvine at Irvine, CA, USA

* Correspondence: Zhi-Ling Guo, MD, PhD

Email: zguo@hs.uci.edu

**Supplementary Tables**

**Supplementary Table 1.** Respiratory rate and temperature following the intervention in the SG.

SG Injection Respiratory Rate (BrPM) Temperature (°C)

Before After Before After

Right side

Normal saline (n=6) 45 ± 5 42 ± 9 36.22 ± 0.32 36.25 ± 0.38

Lidocaine (n=8) 54 ± 19 56 ± 13 36.21 ± 0.34 36.19 ± 0.37

Left side

Normal saline (n=6) 54 ± 5 55 ± 16 36.52 ± 0.46 36.57 ± 0.46

Lidocaine (n=7) 53 ± 15 58 ± 13 36.44 ± 0.39 36.49 ± 0.38

Note: Data are expressed as means ± SD. Comparisons between the two groups were statistically analyzed using the Student's t-test and Mann–Whitney rank sum test. There was no significant difference in respiratory rate and temperature before and after administering 1.0-1.5% lidocaine or 0.9% normal saline on the same right or left side. Also, there was no significant difference between the right and left sides before any treatment. BrPM represents breaths per minute.

**Supplementary Table 2.** Ptosis following the intervention in the SG.

Right eye Left eye

SG Injection Grade Lasting time Grade Lasting time

(range) (min) (range) (min)

Right side

Normal saline (n=6) 0 0 ± 0 0 0

Lidocaine (n=8) 2 - 4 18 ± 4 0 0

Left side

Normal saline (n=6) 0 0 ± 0 0 0

Lidocaine (n=7) 0 0 2 – 4 38 ± 5

Note: Ptosis grade is justified as the percentage of eyelid drooping: 1, 2, 3, and 4 represent 25%, 50%, 75%, and 100% of eyelid drooping, respectively. The 100% of eyelid drooping is the same as eyelid closed.
