# Supplementary material for: Stellate ganglion blockade under ultrasound-guidance and the physiological responses in the rat
Source: Front Physiol. 2025 Jan 10;15:1505038. doi: 10.3389/fphys.2024.1505038 (PMC11757301; doi:10.3389/fphys.2024.1505038)
Supplement: Supplementary file 2 [file DataSheet1.pdf]

Supplementary Materials

Stellate Ganglion Blockade under Ultrasound-Guidance and the Physiological Responses in the Rat

Robert M. N. Tran[1], Shaista Malik[1], Christopher Reist[2, 3], Chad K. Oh[2], Najeebah Abdul-Musawir[2], Stephanie C. Tjen-A-Looi[1], Liang Wu Fu[1], Theodore J. Baird[2], Anh T. Nguyen[1], Yiwei D. Gong[1], Zhi-Ling Guo[1]*

[1]Susan Samueli Integrative Health Institute and Department of Medicine, University of California, Irvine at Irvine, CA, USA
[2]AEON Biopharma, Inc., Irvine, CA, USA
[3]Department of Psychiatry, School of Medicine, University of California, Irvine at Irvine, CA, USA

* Correspondence: Zhi-Ling Guo, MD, PhD
Email: zguo@hs.uci.edu

Supplementary Tables

Supplementary Table S1. Respiratory rate and temperature following the intervention in the SG.

SG Injection	Respiratory Rate (BrPM)		Temperature (°C)	
	Before	After	Before	After
Right side				
Normal saline (n=6)	45 ± 5	42 ± 9	36.22 ± 0.32	36.25 ± 0.38
Lidocaine (n=8)	54 ± 19	56 ± 13	36.21 ± 0.34	36.19 ± 0.37
Left side				
Normal saline (n=6)	54 ± 5	55 ± 16	36.52 ± 0.46	36.57 ± 0.46
Lidocaine (n=7)	53 ± 15	58 ± 13	36.44 ± 0.39	36.49 ± 0.38

Note: Data are expressed as means ± SD. Comparisons between the two groups were statistically analyzed using the Student's t-test and Mann–Whitney rank sum test. There was no significant difference in respiratory rate and temperature before and after administering 1.0-1.5% lidocaine or 0.9% normal saline on the same right or left side. Also, there was no significant difference between the right and left sides before any treatment. BrPM represents breaths per minute.

Supplementary Table S2. Ptosis following the intervention in the SG.

SG Injection	Right eye		Left eye	
	Grade (range)	Lasting time (min)	Grade (range)	Lasting time (min)
Right side				
Normal saline (n=6)	0	0 ± 0	0	0
Lidocaine (n=8)	2 - 4	18 ± 4	0	0
Left side				
Normal saline (n=6)	0	0 ± 0	0	0
Lidocaine (n=7)	0	0	2 – 4	38 ± 5

Note: Ptosis grade is justified as the percentage of eyelid drooping: 1, 2, 3, and 4 represent 25%, 50%, 75%, and 100% of eyelid drooping, respectively. The 100% of eyelid drooping is the same as eyelid closed.
